# CTRP3 attenuates myocardial lipotoxicity via suppression of lipid accumulation, inflammation, apoptosis, and mitochondrial oxidative stress

**DOI:** 10.3389/fcvm.2025.1575929

**Published:** 2025-05-13

**Authors:** Qingpeng Wang, Jiangyang Chi, Chen Wang, Yanhong Yuan, Rui Tian, Yun Yang, Xinzhong Chen

**Affiliations:** ^1^ Department of Cardiovascular Surgery, Union Hospital, Tongji Medical College, Huazhong University of Science and Technology, Wuhan, China; ^2^ Department of Ultrasound Medicine, Union Hospital, Tongji Medical College, Huazhong University of Science and Technology, Wuhan, China

**Keywords:** CTRP3, myocardial lipotoxicity, lipid metabolism, inflammation, oxidative stress

## Abstract

Myocardial lipotoxicity, a pathophysiological condition characterized by cardiomyocyte damage resulting from dysregulated fatty acid metabolism, plays a pivotal role in cardiovascular disease progression. C1q/tumor necrosis factor-related protein-3 (CTRP3), a novel adipocytokine with pleiotropic metabolic regulatory properties, has recently been implicated in lipid homeostasis modulation. Nevertheless, its cardioprotective potential against myocardial lipotoxicity remains poorly understood.

**Objective:**

A comprehensive approach combining *in vivo* high-fat diet (HFD) murine models and *in vitro* palmitic acid-induced cardiomyocyte injury systems was employed.

**Methods:**

this study used animal and cellular experiments to verify the function of CTRP3.

**Results:**

HFD feeding induced significant lipid droplet deposition in cardiomyocytes, concomitant with enhanced inflammatory responses, elevated apoptotic activity, and exacerbated oxidative stress, ultimately leading to cardiac dysfunction. Both cardiac-specific CTRP3 overexpression and exogenous recombinant CTRP3 (rCTRP3) administration demonstrated remarkable cardioprotective effects, manifested through: (1) Significant attenuation of intramyocardial lipid accumulation (*p* < 0.05) (2) Suppression inflammatory pathways (3) Inhibition of mitochondrial-dependent apoptosis (4) Enhancement of antioxidant defense systems. These coordinated effects substantially ameliorated lipotoxic myocardial damage and improved cardiac functional parameters.

**Conclusion:**

Our findings reveal that CTRP3 confers robust protection against myocardial lipotoxicity through multi-modal mechanisms involving lipid metabolism regulation, anti-inflammatory actions, apoptosis inhibition, and oxidative stress mitigation, highlighting its therapeutic potential for metabolic cardiomyopathy.

## 1 Introduction

The global epidemic of obesity is leading to a concerning rise in the incidence and associated risks of coronary heart disease (1). Obesity and prolonged high-fat diets not only contribute to the narrowing of coronary arteries but also disrupt energy metabolism within myocardial cells and tissues, resulting in significant myocardial ischemia (2, 3). Individuals with obesity experience cardiovascular diseases at a younger age, facing a risk 2–4 times greater than their non-obese counterparts and often exhibiting a poorer prognosis (4). Dysregulation of glucose and lipid metabolism is a critical factor in the development of myocardial lipotoxicity (5). Conditions such as type 2 diabetes, hyperlipidemia, insulin resistance, and systemic inflammation are key contributors to myocardial dysfunction, which negatively impacts patients’ daily activities and overall health (6). Even before overt heart dysfunction manifests through symptoms such as myocardial ischemia, angina, or heart attacks, significant alterations are already occurring within the cellular and tissue architecture of the myocardium (7). Disordered lipid metabolism leads to excessive lipid deposition within myocardial cells and tissues, producing toxic lipid intermediates that exacerbate myocardial lipotoxicity. This, combined with reduced ATP generation, compromises the heart's ability to contract and relax, ultimately resulting in myocardial dysfunction (8–11). Therefore, targeting the prevention or amelioration of myocardial ischemia through the lens of myocardial lipid metabolism is of paramount importance (12). Investigating the mechanisms by which obesity or a prolonged high-fat diet induces myocardial lipotoxicity and identifying potential therapeutic targets to alleviate this condition and improve cardiac function hold immense scientific and clinical significance.

Myocardial lipotoxicity is a pathological phenomenon characterized by excessive lipid accumulation in non-adipose tissues, which disrupts myocardial lipid metabolism and impairs the heart's contraction and relaxation functions (8, 13). As myocardial lipotoxicity progresses, myocardial dysfunction also worsens. Therefore, it is essential to identify the micro-level aspects and mechanisms that influence myocardial lipid metabolism in the early stages to improve myocardial lipotoxicity (14). C1q/TNF-related protein (CTRP) is a highly conserved family of 15 members, each with distinct structures and functions, recognized as novel secreted metabolic regulators reminiscent of the adiponectin globular domain (15). Among them, CTRP3 is an important adipokine recently identified as being secreted by adipocytes and myocardial cells, among others. CTRP3 is highly expressed in white adipose tissue, as well as in the heart and liver (15). It circulates in the blood and functions similarly to endocrine hormones, with a serum concentration of approximately 1 ± 0.3 mg/ml (16). Research has shown that CTRP3 can regulate hepatic glucose output, alleviate liver lipid degeneration, inhibit inflammatory responses, and improve liver lipid metabolism (17, 18). In myocardial cells, downregulation of CTRP3 can induce inflammatory responses and cell apoptosis (19). Following myocardial infarction (MI), the expression of CTRP3 significantly decreases in mouse plasma and myocardium. Supplementation with recombinant CTRP3 has been found to improve survival rates in mice, reduce myocardial hypertrophy, and decrease fibroblast proliferation post-MI (20–22). However, the role of CTRP3 in myocardial lipid metabolism remains unclear, and the potential mechanisms underlying its biological function have yet to be elucidated. This study aims to further clarify these mechanisms.

## 2 Methods

### 2.1 Animals

Male ob/ob mice (8 weeks old, *n* = 30) were procured from Jiangsu Jicui Pharmaceutical Co., Ltd. (Nanjing, China) and acclimatized under specific pathogen-free (SPF) conditions (12 h light/dark cycle, 22 ± 1°C, 50% humidity) for 1 week. Mice were randomized into three groups: Control (regular chow), HFD (60% fat diet), and HFD + rCTRP3 (100 μg/kg/day intraperitoneal injection). This study has obtained ethical approval from the Ethics Committee of Huazhong University of Science and Technology Tongji Medical College.

### 2.2 Cardiac functional assessment

All experimental subjects were anesthetized with isoflurane (2.5%–3.5% v/v) and maintained under physiological monitoring during echocardiographic evaluation. Cardiac function was systematically analyzed using a high-resolution ultrasound imaging system (Vevo® 3,100, VisualSonics, Toronto, Canada) with a 21-MHz linear array transducer. Standard parasternal long-axis views were acquired to quantify:

• Left ventricular end-diastolic volume (LVEDV)
• Left ventricular end-systolic volume (LVESV)
• Left ventricular internal diameter at end-diastole (LVIDd)
• Left ventricular internal diameter at end-systole (LVIDs)

Functional parameters including left ventricular ejection fraction (LVEF) and fractional shortening (FS) were calculated using the following formulae:

LVEF(%)=(LVEDV−LVESV)/LVEDV×100

FS(%)=(LVIDd−LVIDs)/LVIDd×100

All measurements were performed by blinded operators and averaged across three consecutive cardiac cycles to ensure data reproducibility.

### 2.3 rCTRP3

The recombinant CTRP3 protein (rCTRP3, Catalog Number: CSB-EP883621HU) was commercially procured from Wuhan Cusabio Biotech Co., Ltd. (Hubei, China). The lyophilized protein was reconstituted in enzyme-free water. The rCTRP3 was subsequently administered to ob/ob mice through intraperitoneal injection at a dose of 100 μg/ml/animal/day, with a volume of 200 μl per injection (17, 23). The injections were performed every other day. The animal experiments were approved by the ethics committee.

### 2.4 Lipid testing

Myocardial tissue samples were freshly collected and immediately fixed in 4% paraformaldehyde solution. Subsequently, frozen sections were prepared from the fixed tissues. Oil Red staining was applied to visualize lipid droplets within the myocardial cells. The number and size of these lipid droplets were meticulously examined and quantified using a microscope (Carl Zeiss, Germany).

### 2.5 Histopathological examination

HE and Masson staining were performed to assess the cardiac structure and degree of fibrosis. Images were acquired using a microscope (Carl Zeiss, Germany).

### 2.6 Elisa

Animal blood samples were meticulously collected and subsequently centrifuged at 1,000*g* for 20 min. The supernatant was carefully aspirated and stored at −80°C to preserve its integrity until further analysis. The concentrations of triglycerides (TG), total cholesterol (TCHO), tumor necrosis factor-alpha (TNFα), interleukin-1β (IL1β), interleukin-6 (IL-6), B-cell lymphoma 2 (Bcl2), Bcl-2 associated X protein (Bax), superoxide dismutase 1 (SOD1), and superoxide dismutase 2 (SOD2) in the serum were determined using specific enzyme immunoassay kits (HY20852, HY20188, HY20174, HYRX200327, HYRX200325, HY-0306M1, HY-BL1250, HYSH467, HYSH469, sourced from Wuhan, China). All measurements were carried out in strict accordance with the manufacturer's instructions to ensure accuracy and reproducibility.

### 2.7 Immunofluorescence

Frozen myocardial sections were retrieved from −80°C storage and prepared for fluorescence staining. The staining procedure involved the sequential application of primary antibodies, fluorescent quenching agents, secondary antibodies, and DAPI (4’,6-diamidino-2-phenylindole) for nuclear counterstaining. The stained myocardial tissue sections were then examined under a fluorescence microscope (Carl Zeiss, Germany) to observe the fluorescence intensity. High-resolution images were captured to document the staining patterns and intensity for further analysis.

### 2.8 DHE staining

To assess the production of reactive oxygen species (ROS), dihydroethidium (DHE) staining was performed on left ventricular tissue sections. The sections were incubated with a DHE solution (5 μmol/L; Beyotime Biotechnology, Shanghai, China) for 30 min at 37°C in a humidified chamber. Following incubation, the fluorescence intensity, indicative of superoxide production, was examined and imaged using a fluorescence microscope (Carl Zeiss, Germany). The intensity of the red fluorescence was quantified to evaluate the level of ROS generation in the myocardial tissue.

### 2.9 RNA-seq

Myocardial tissues were freshly collected from mice in the control group, high-fat group, and CTRP3 group. Total RNA was extracted from these tissues and subsequently sent to BGI Genomics for comprehensive transcriptome sequencing analysis to elucidate the gene expression profiles in the myocardium.

### 2.10 q-PCR

Total RNA extraction from myocardial cells was conducted using the Fast Cell or Tissue Total RNA Isolation Kit (Catalog Number: RC112-01) from Novogene Biotechnology Co., Ltd. (Nanjing, China), in strict accordance with the manufacturer's instructions. The sequences of all primers used for quantitative polymerase chain reaction (qPCR) are provided in Supplementary Table S1.

### 2.11 Cell culture

The H9c2 myocardial cell line was obtained from Wuhan PunoSai Company. Neonatal rat cardiomyocytes (NRCMs) were isolated from the hearts of 1- to 3-day-old Sprague-Dawley (SD) rats. The isolated cells were digested with trypsin and subsequently cultured in high-glucose DMEM medium supplemented with 10% fetal bovine serum (FBS). The cells were maintained in an incubator at 37°C with a humidified atmosphere of 95% O₂ and 5% CO₂.

### 2.12 Plasmatic acid (PA)

The palmitic acid (PA) solution was purchased from Kunchuang Biotechnology (Xi’an, China; Catalog Number: SYSJ-KJ003/KC003). The PA was prepared at various concentrations and added to the cells when they were in a healthy growth state.

### 2.13 CCK8

A CCK8 working solution was prepared by mixing the CCK8 solution with high-glucose DMEM medium at a ratio of 1:10. The cells were subsequently incubated with this working solution at 37°C in a humidified incubator with 95% O₂ and 5% CO₂ for 1.5 h. The absorbance was then measured at 450 nm to assess cell viability.

### 2.14 Transmission electron microscopy (TEM)

Fresh cardiomyocytes were collected and immediately fixed in 2.5% glutaraldehyde solution, followed by storage at 4°C. Ultrastructural images of the cardiomyocytes were captured using a Hitachi H-7000 transmission electron microscope (Pleasanton, CA, USA). Mitochondrial morphology was evaluated using ImageJ software. Mitochondrial damage was defined by the presence of disrupted mitochondrial cristae and irregular mitochondrial arrangement. The ratio of damaged mitochondria to total mitochondria was quantified to assess the extent of mitochondrial injury.

### 2.15 Mitochondrial detection

The staining of cardiomyocyte mitochondria was performed using JC-1 and Mito-Tracker dyes. The stained cells were then examined under a fluorescence microscope (Carl Zeiss, Germany) to assess mitochondrial number and membrane potential.

### 2.16 ROS testing

The DCFH-DA dye was diluted in high-glucose DMEM or 1 × PBS at a ratio of 1:1000, resulting in a working concentration of 10 μM. The cells were then incubated in a CO₂ incubator at 37°C for 0.5 h. After the incubation period, the cells were collected, and fluorescent signals indicative of reactive oxygen species (ROS) were measured using either a laser confocal microscope or flow cytometer.

### 2.17 Flow cytometry

To assess apoptosis in cardiomyocytes, the cells were first centrifuged at 1,000*g* for 5 min using a low-speed centrifuge, and the supernatant was carefully removed. Each cell pellet was then resuspended in 195 μl of Annexin V-FITC binding buffer. Subsequently, 5 μl of propidium iodide (PI) staining solution was added to each tube, gently mixed, and incubated on ice in the dark. Flow cytometry was performed to detect the fluorescence signals, with Annexin V-FITC emitting green fluorescence and PI emitting red fluorescence.

### 2.18 Statistical analysis

In this study, data analysis was conducted using a combination of software tools, including GraphPad Prism 9.0 for graphing and statistical analysis, SPSS 8.0 for advanced statistical modeling, ImageJ for image analysis, and Photoshop 2023 for image processing and figure preparation. For statistical analysis, independent sample *t*-tests were employed to compare data between two groups, while one-way or two-way analysis of variance (ANOVA) was used to compare data among multiple groups. The results are presented as mean ± standard deviation (mean ± SD). Statistical significance was defined as *p* < 0.05, and significant differences are indicated with asterisks (*) or other appropriate notations in the figures and tables.

## 3 Results

### 3.1 High-fat diet induces myocardial lipid accumulation and dysfunction

**Figure 1:**
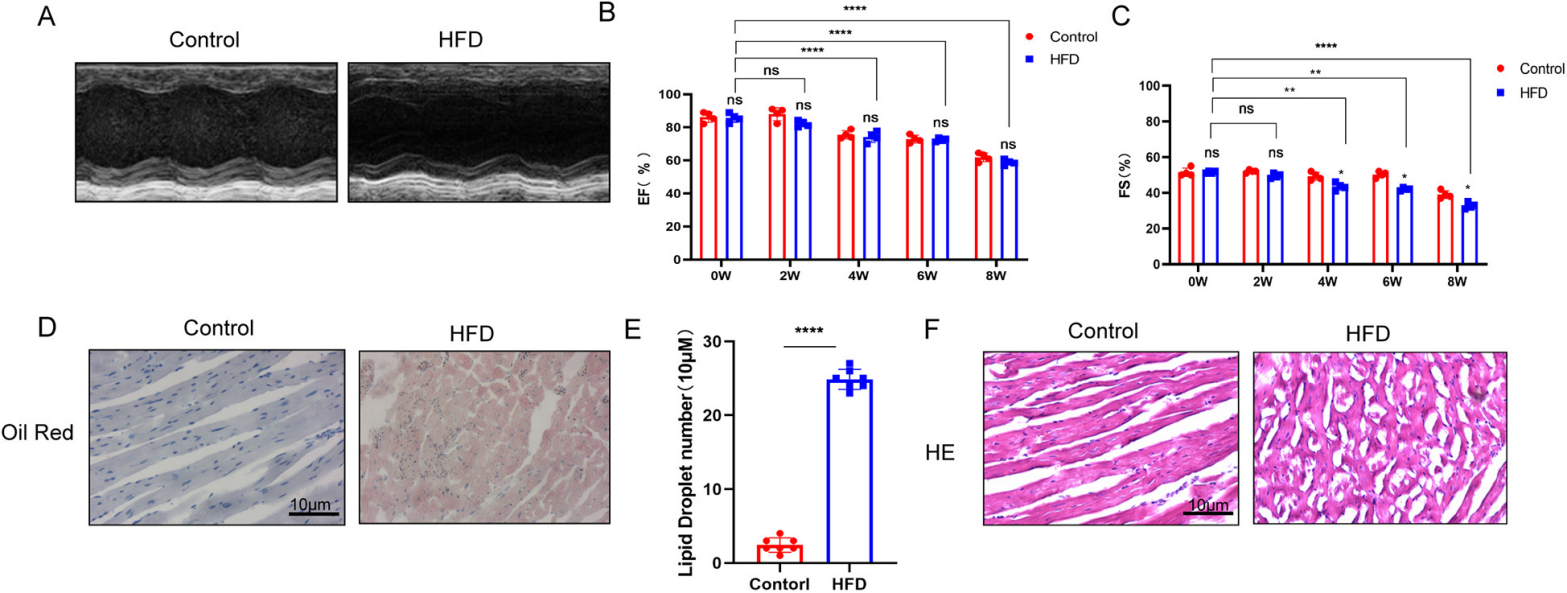
**(A–C)** Cardiac ultrasound results demonstrate that a high-fat diet progressively impairs cardiac function over time (*n* ≥ 3, *p* < 0.05). **(D,E)** Oil Red O staining reveals significant accumulation of lipid droplets in myocardial tissue following a high-fat diet. **(F)** Hematoxylin and eosin (H&E) staining indicates that a high-fat diet induces structural disarray in myocardial tissue (Supplementary Figure S1).

In this study, ob/ob mice were assigned to two dietary groups: one group was fed a standard chow diet, while the other group was fed a high-fat diet (60% fat, 20% protein, 20% carbohydrates). After 8 weeks of dietary intervention, mice in the high-fat diet group exhibited significant increases in body weight and developed obesity, alongside reduced physical activity. And as the duration of a high-fat diet increases, cardiac function gradually declines (Figure 1A–C). Histological examination of myocardial tissue revealed substantial accumulation of lipid droplets in the hearts of these mice. Additionally, cardiac function was significantly impaired in the high-fat diet group, as evidenced by decreased cardiac activity. In contrast, mice fed the standard chow diet showed no evidence of myocardial lipid accumulation and maintained normal cardiac function (Figure 1D–F). These results indicate that chronic consumption of a high-fat diet promotes lipid accumulation in the myocardium, thereby contributing to cardiac dysfunction in mice.

### 3.2 High-fat diet causes myocardial cell inflammation, apoptosis, and oxidative stress

**Figure 2:**
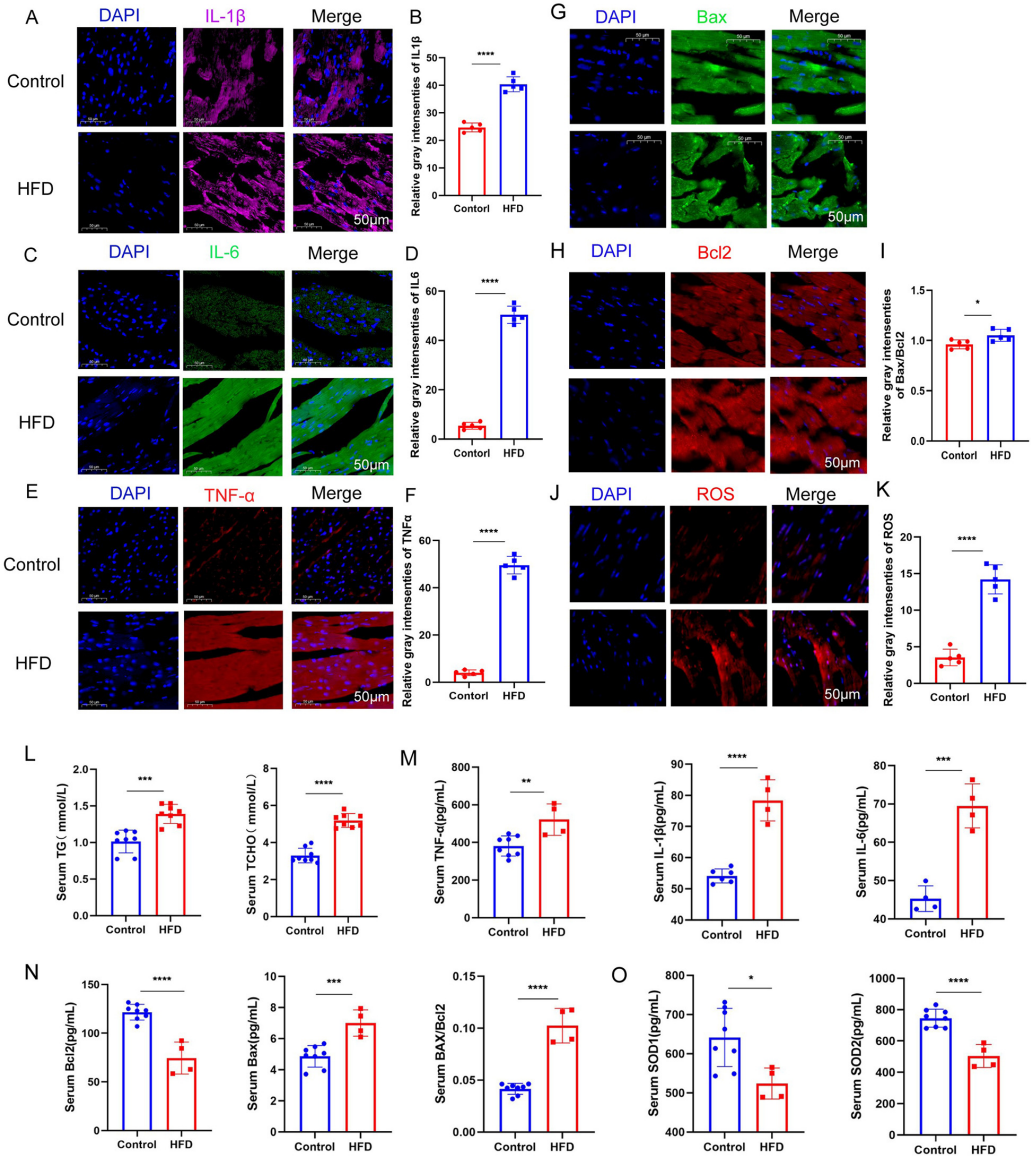
**(A–F)** Immunofluorescence staining results demonstrate that a high-fat diet significantly enhances the inflammatory response in myocardial tissue. **(G–K)** Additionally, a high-fat diet induces increased myocardial apoptosis and oxidative stress response, as evidenced by elevated expression of related markers. **(L–O)** Serum ELISA assays reveal that a high-fat diet leads to significant increases in the levels of triglycerides, cholesterol, inflammatory factors, apoptotic factors, and oxidative stress markers (*n* ≥ 3, *p* < 0.05).

In the myocardial tissue of ob/ob mice fed a high-fat diet, we observed significant increases in the levels of inflammatory cytokines, including IL-6, IL-1β, and TNF-α (Figure 2A–F). Additionally, the ratio of apoptotic markers Bax/Bcl-2 was elevated, indicating increased apoptosis (Figure 2G–I). Oxidative stress levels were also significantly higher in the myocardial tissue of these mice (Figure 2J,K). In contrast, myocardial tissue from the control group fed a regular diet exhibited no significant changes in inflammatory markers, apoptotic indices, or oxidative stress levels. Serum ELISA assays reveal that a high-fat diet leads to significant increases in the levels of triglycerides, cholesterol, inflammatory factors, apoptotic factors, and oxidative stress markers (Figure 2L–O). These findings suggest that a prolonged high-fat diet induces myocardial cell inflammation, apoptosis, and oxidative stress, which are hallmarks of myocardial lipotoxicity.

### 3.3 Palmitic acid (PA) induces lipid accumulation in myocardial cells

**Figure 3:**
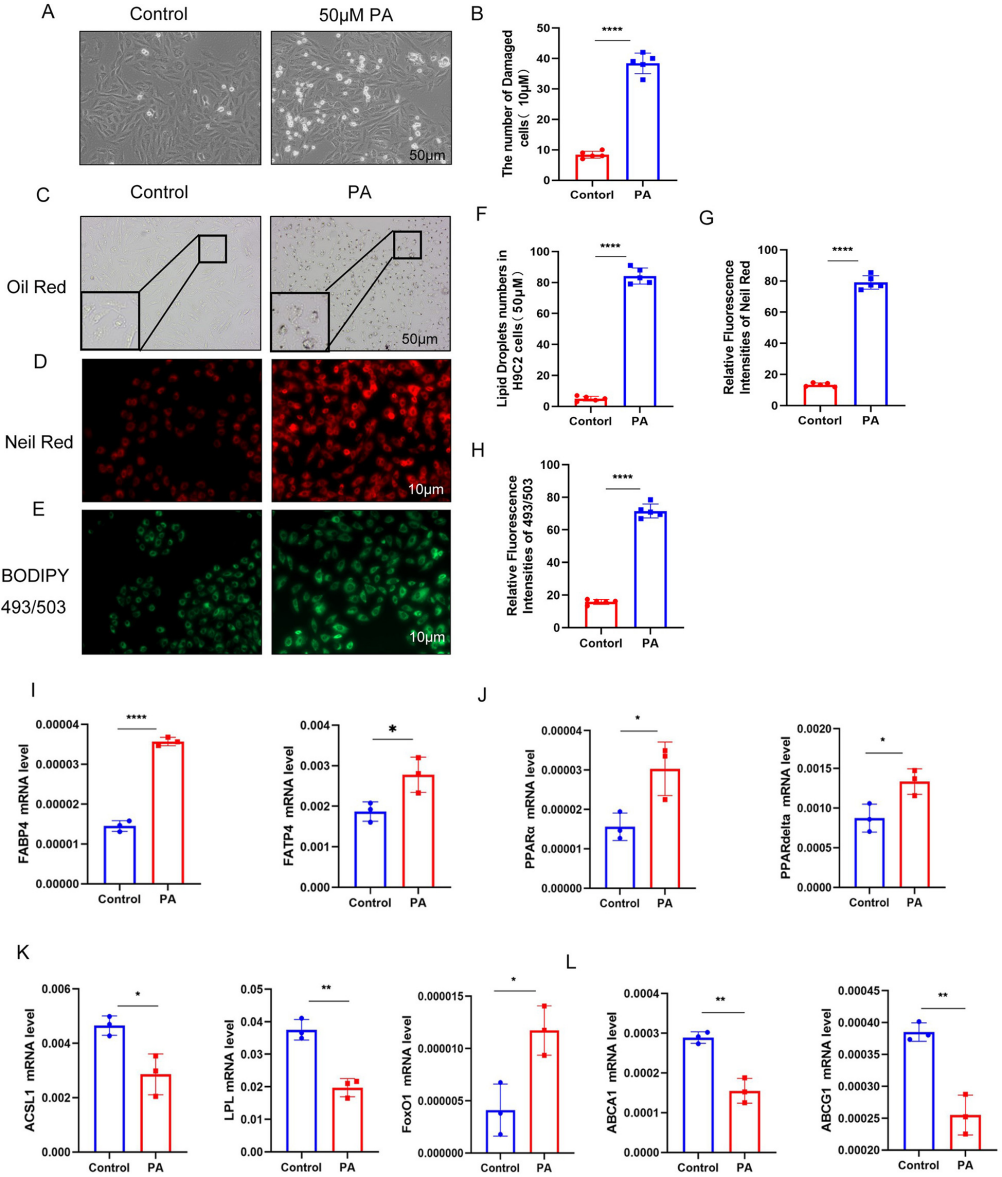
**(A,B)** Compared to the control group, myocardial cell damage was significantly increased following palmitic acid (PA) intervention. **(C–H)** Lipid testing results demonstrate a substantial accumulation of lipid droplets in myocardial cells after PA intervention compared to the control group. **(I–L)** After PA intervention, the expression of genes involved in fatty acid uptake was significantly upregulated, while the expression of genes related to fatty acid breakdown was downregulated. Additionally, the expression of lipid efflux genes was also decreased (*n* ≥ 3, *p* < 0.05).

In this study, we treated H9c2 myocardial cells with 50 μM palmitic acid (PA) for 72 h to establish an *in vitro* model of myocardial lipotoxicity (Supplementary Figure S2). The control group was treated with high-glucose DMEM supplemented with PBS, while the experimental group received high-glucose DMEM supplemented with PA, thereby simulating the effects of a high-fat environment on myocardial cells (Figure 3A,B).

Oil Red O staining revealed no significant lipid droplet accumulation in the myocardial cells of the control group, whereas the experimental group exhibited substantial lipid droplet accumulation. Consistent results were obtained using Nile Red staining and BODIPY 493/503 neutral lipid detection, further confirming the increased lipid accumulation in the experimental group (Figure 3C–H). These findings suggest that prolonged exposure to high-fat conditions can disrupt myocardial lipid metabolism, leading to the accumulation of lipid droplets within myocardial cells.

Additionally, in the myocardial tissue, the levels of fatty acid transport protein 4 (FATP4), fatty acid binding protein 4 (FABP4), and very low-density lipoprotein receptor (VLDLR) were significantly increased. The levels of peroxisome proliferator-activated receptors α and δ (PPARα/PPARδ) were also elevated, indicating an upregulation of pathways involved in fatty acid uptake and storage. Conversely, the levels of genes related to fatty acid catabolism and utilization, such as ASCL1, LPL, and FoxO1, were increased, while the levels of cholesterol efflux proteins (ABCA1 and ABCG1) were decreased (Figure 3I–L). These changes collectively suggest that prolonged high-fat treatment can alter the expression of key regulators of lipid metabolism, contributing to lipid accumulation in myocardial cells.

### 3.4 PA induces inflammation, apoptosis, and oxidative stress in myocardial cells

**Figure 4:**
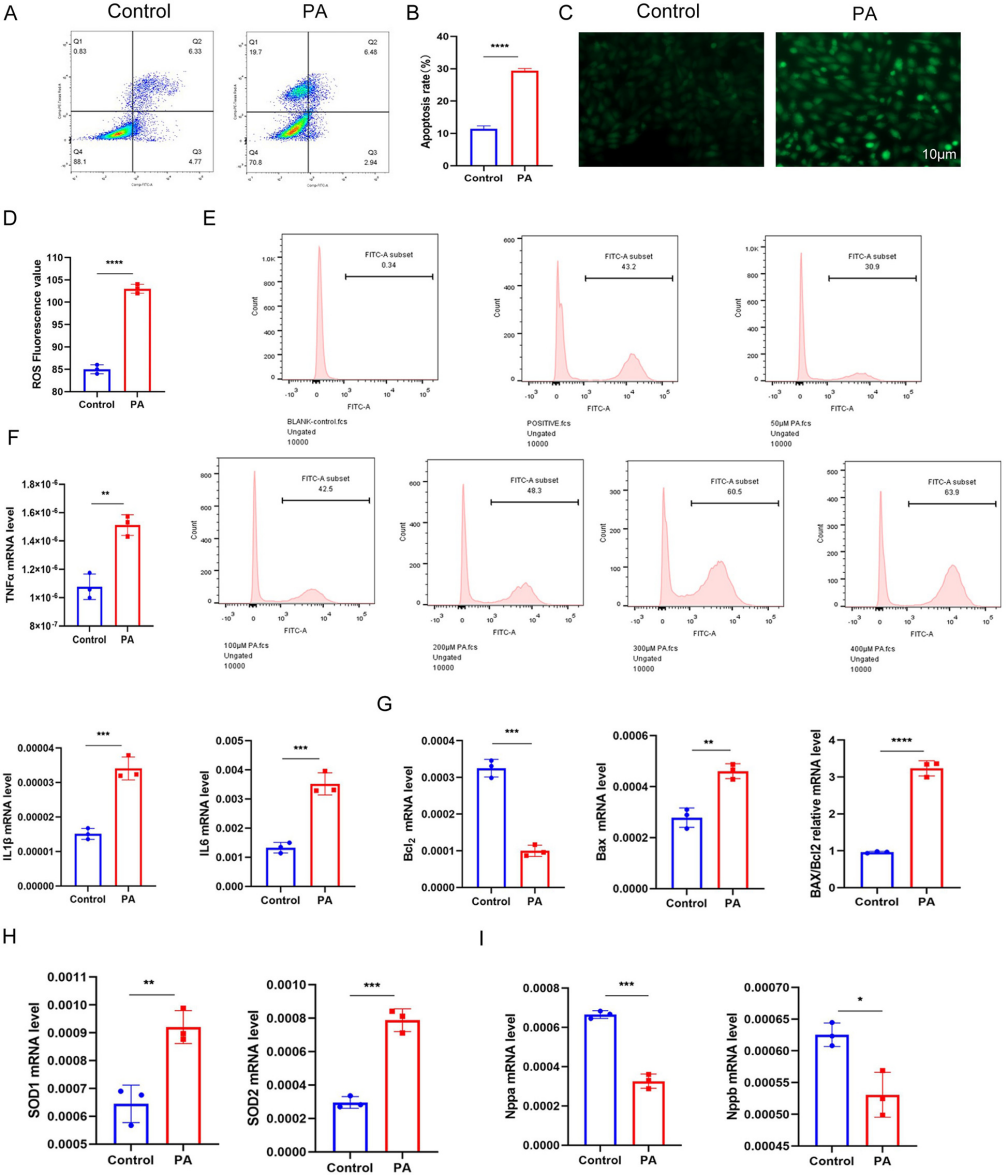
**(A,B)** Flow cytometry results demonstrate a significant increase in apoptotic and necrotic cells in myocardial cells following palmitic acid (PA) intervention. **(C–E)** Immunofluorescence and flow cytometry results indicate elevated levels of oxidative stress in myocardial cells after PA intervention (Supplementary Figure S3). **(F–I)** Quantitative PCR (qPCR) results show upregulation of inflammatory factors, apoptotic factors, and oxidative stress markers in myocardial cells (*n* ≥ 3, *p* < 0.05).

In flow cytometry experiments, the experimental group exhibited a significant increase in both apoptotic and necrotic cells (Figure 4A,B). Additionally, the levels of reactive oxygen species (ROS) in myocardial cells were found to rise in proportion to increasing concentrations of PA. Immunofluorescence assays further confirmed a marked elevation in ROS production within the experimental group (Figure 4C–E).

Concurrently, myocardial tissue from the experimental group showed significant increases in inflammatory mediators, including TNF-α, IL-1β, and IL-6. There were also notable increments in apoptotic markers such as the Bax/Bcl-2 ratio, as well as oxidative stress markers, including SOD1 and SOD2. In contrast, the levels of natriuretic peptides (Nppa and Nppb) were decreased (Figure 4F–I).

These findings indicate that sustained high-fat treatment of myocardial cells results in distinct pathological changes, characterized by enhanced lipid deposition, apoptotic injury, inflammatory responses, and oxidative stress. Collectively, these alterations are indicative of myocardial lipotoxicity, which is defined as the toxic impairment of myocardial cells precipitated by lipid accumulation.

### 3.5 CTRP3 alleviates myocardial lipid accumulation, inflammation, and apoptosis, thereby improving myocardial function

To elucidate the role of the adipokine CTRP3 in myocardial lipotoxicity, this study administered exogenous recombinant CTRP3 (rCTRP3) protein to ob/ob mice fed a high-fat diet. The animal experiment comprised three groups: a control group (Control group) fed a regular diet, a high-fat diet group (HFD group), and a CTRP3 group receiving a high-fat diet supplemented with intraperitoneal injections of rCTRP3 at a dose of 100 μg/ml/animal/day (17, 23). All mice were maintained under identical housing conditions, and their myocardial status and function were monitored.

**Figure 5:**
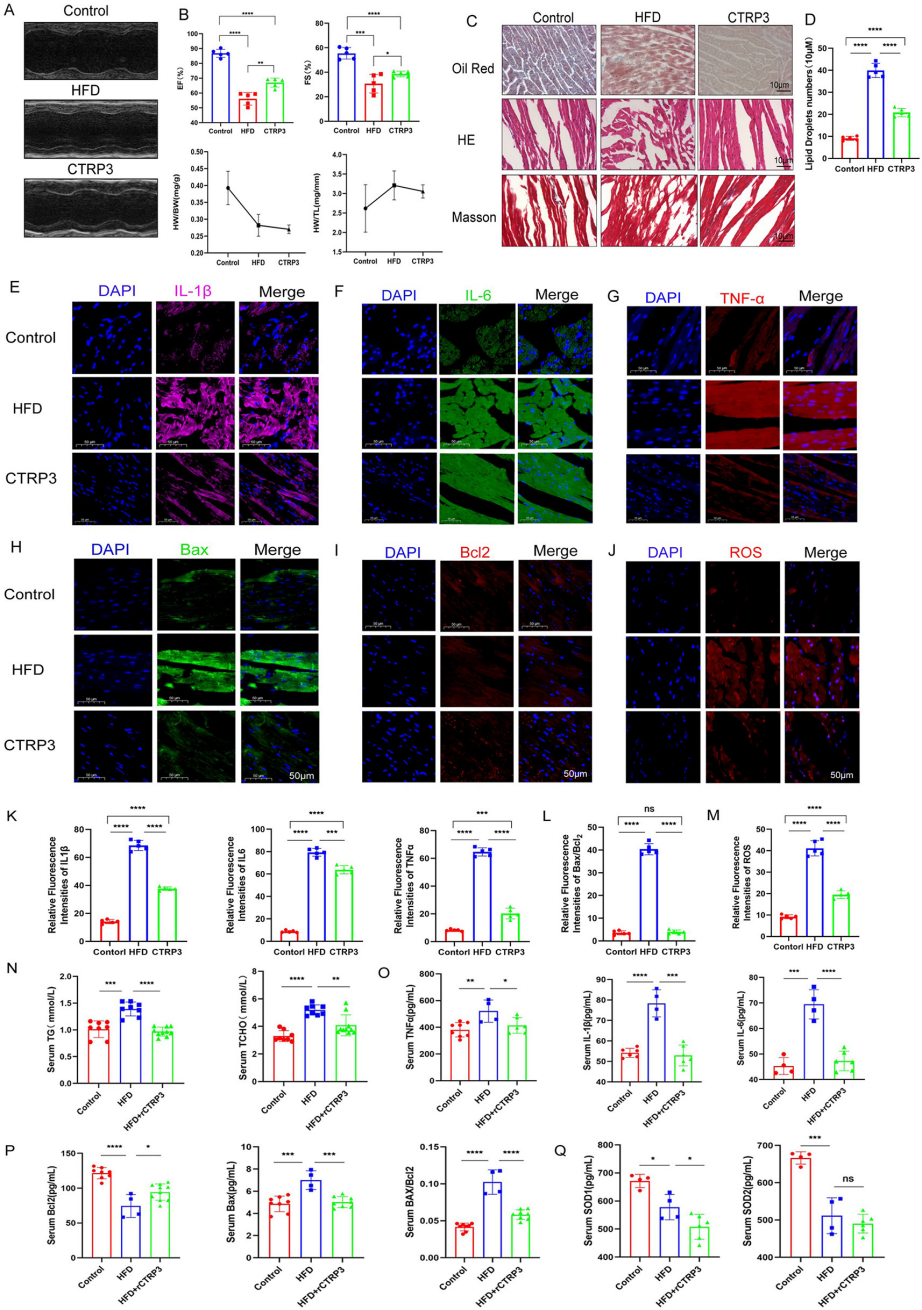
**(A,B)** Compared to the high-fat diet (HFD) group, supplementation with CTRP3 significantly improves cardiac function in mice, as evidenced by enhanced left ventricular ejection fraction. **(C,D)** Histological examination of myocardial tissue reveals substantial lipid droplet accumulation in the HFD group. However, CTRP3 supplementation reduces lipid droplet accumulation, improves myocardial tissue structure, and alleviates the degree of fibrosis. **(E–M)** Immunofluorescence results indicate that CTRP3 supplementation mitigates myocardial inflammation, apoptosis, and oxidative stress in mice. **(N–Q)** Serum ELISA assays demonstrate that levels of triglycerides (TG), total cholesterol (TCHO), inflammatory markers, apoptotic factors, and oxidative stress indicators are significantly improved in the CTRP3 group compared to the HFD group (*n* ≥ 3, *p* < 0.05).

The results demonstrated that, compared to the high-fat diet group, rCTRP3 supplementation significantly improved cardiac function in the animals, as evidenced by a marked increase in left ventricular ejection fraction (Figure 5A,B). Moreover, myocardial tissue from rCTRP3-treated mice exhibited reduced lipid droplet accumulation (Figure 5C,D), decreased levels of inflammatory and apoptotic markers (including IL1βR1 and IL1α levels under high-fat conditions by CTRP3 treatment was also verified in Supplementary Figure S6), and lower oxidative stress levels (Figure 5E–M). Serum ELISA assays demonstrate that levels of triglycerides (TG), total cholesterol (TCHO), inflammatory markers, apoptotic factors, and oxidative stress indicators are significantly improved in the CTRP3 group compared to the HFD (Figure 5N–Q). These findings suggest that CTRP3 can mitigate the adverse effects of a high-fat diet on myocardial cells, thereby improving myocardial function in obese ob/ob mice.

### 3.6 CTRP3 alleviates myocardial cell lipid accumulation, inflammation, apoptosis, and oxidative stress

To investigate the role of the adipokine CTRP3 in myocardial cell lipotoxicity, we conducted a cell experiment with three groups: a control group (Control) treated with high-glucose DMEM + PA, an experimental group (PA) treated with high-glucose DMEM + PA, and an intervention group (PA + CTRP3) treated with high-glucose DMEM + PA + 10 μg/ml recombinant CTRP3 (rCTRP3) (Supplementary Figure S4).

**Figure 6:**
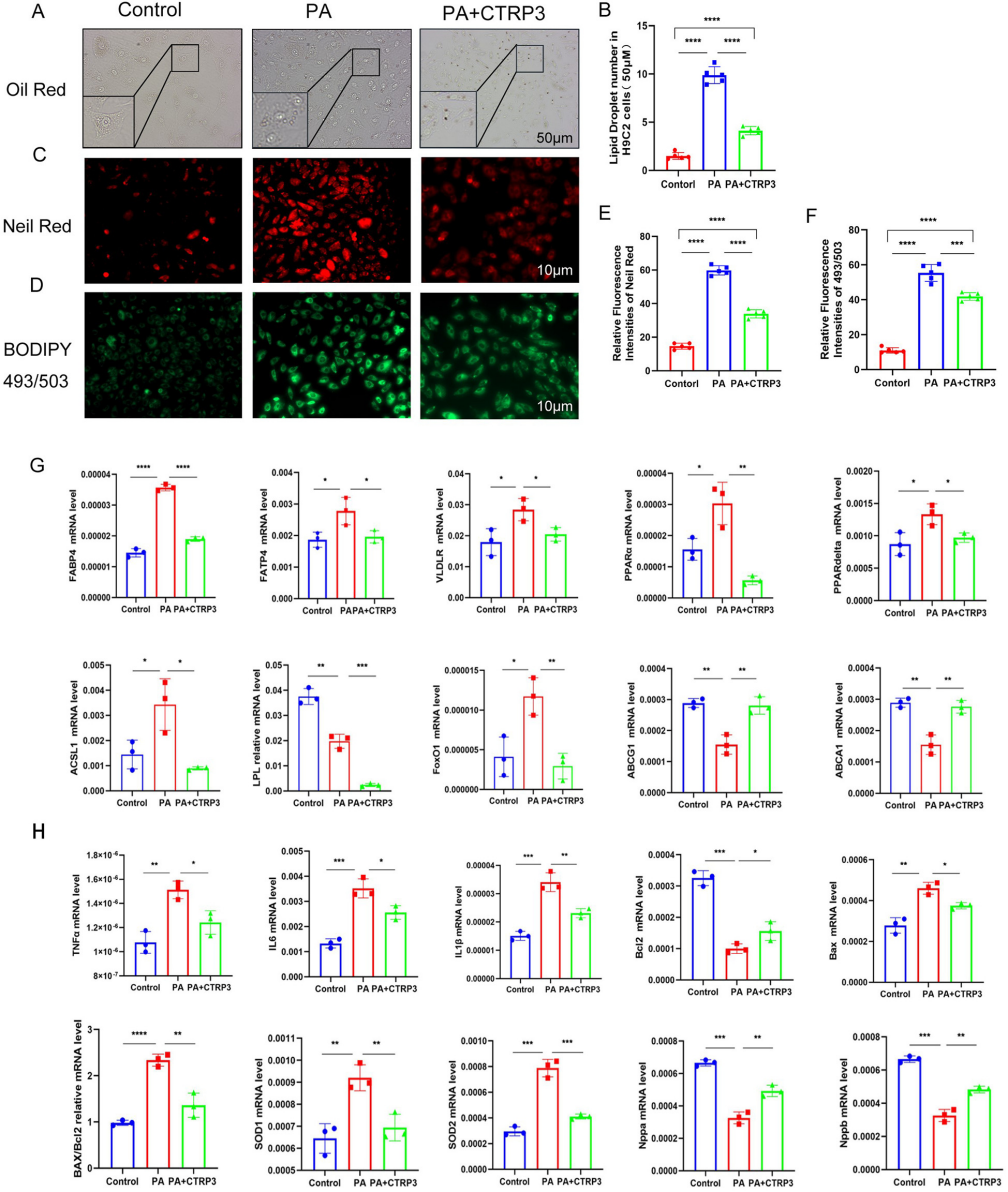
**(A–F)** Lipid testing results demonstrate that palmitic acid (PA) induces lipid accumulation in myocardial cells, while CTRP3 supplementation significantly reduces lipid droplet accumulation. **(G)** CTRP3 intervention improves the expression of genes related to fatty acid uptake, fatty acid oxidation, and lipid efflux in myocardial cells (*n* ≥ 3, *p* < 0.05). **(H)** Quantitative PCR (q-PCR) results indicate that CTRP3 mitigates inflammation, apoptosis, and oxidative stress in myocardial cells stimulated by PA (*n* ≥ 3, *p* < 0.05).

The results showed a significant reduction in lipid droplet accumulation in myocardial cells following rCTRP3 intervention (Figure 6A,B). Nile Red staining and BODIPY 493/503 neutral lipid staining further confirmed the decreased lipid droplet content in the intervention group (Figure 6C–F). These findings suggest that prolonged high-fat stimulation disrupts myocardial lipid metabolism, leading to an imbalance between lipid uptake and oxidative utilization, and consequently, the accumulation of lipid droplets in myocardial cells. High-fat stimulation also affects epicardial and systemic adipose tissue, altering the secretion of adipokines, including CTRP3. Our results indicate that CTRP3 may play a role in mitigating key aspects of myocardial lipid metabolism and improving myocardial cell function. Additionally, genes involved in lipid metabolism exhibited corresponding changes (Figure 6G), suggesting that CTRP3 may reduce myocardial lipid accumulation by promoting cholesterol and lipid efflux and decreasing fatty acid uptake.

In myocardial cells with lipotoxicity, CTRP3 intervention significantly improved inflammation, apoptotic injury, and oxidative stress (Figure 6H, Supplementary Figures S5, S6). These experimental results demonstrate that CTRP3 not only reduces lipid droplet accumulation in myocardial cells but also alleviates apoptotic injury, oxidative stress, and inflammatory responses, thereby mitigating myocardial lipotoxicity.

### 3.7 CTRP3 is involved in regulating high-fat diet-induced mitochondrial damage in the myocardium

**Figure 7:**
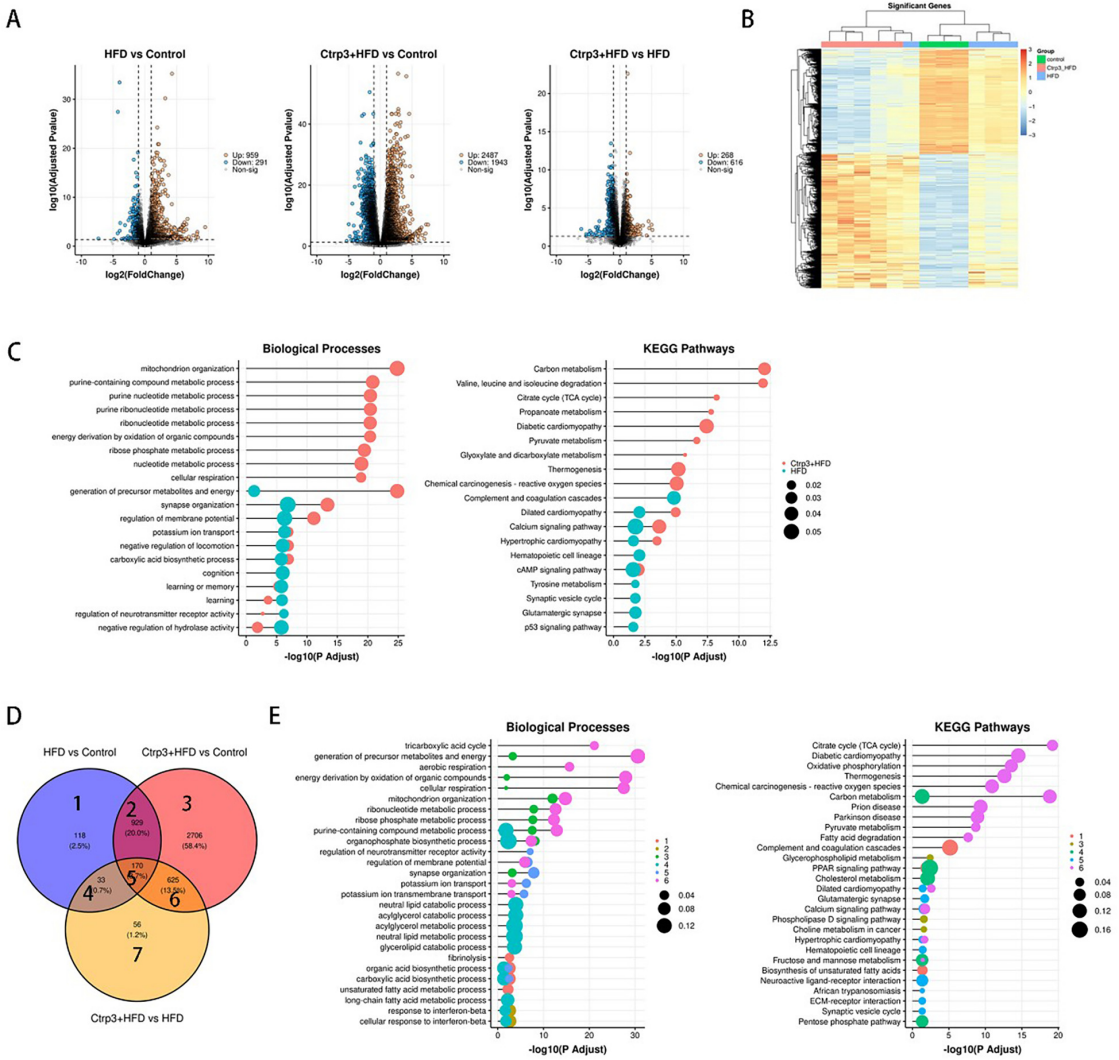
**(A–E)** RNA sequencing analysis of myocardial tissue from the control group, HFD group, and CTRP3 group indicates that CTRP3 likely exerts its effects within myocardial mitochondria, playing a role in the metabolic regulation of myocardial lipotoxicity.

Transcriptomic analysis of myocardial tissue from control, high-fat diet (HFD), and CTRP3 groups revealed significant correlations between genes associated with lipid metabolism, mitochondrial function, and oxidative stress processes and both the HFD and CTRP3 groups (Figure 7A–E). These findings further suggest that CTRP3 may participate in cardiac mitochondrial energy metabolism and transport processes. The study results indicate that high-fat stimulation leads to mitochondrial structural damage in myocardial cells and impairs cardiac function.

Differential gene analysis was performed by comparing the Control group vs. HFD group, Control group vs. CTRP3 group, and HFD group vs. CTRP3 group. Significantly different genes and heatmaps were obtained for each comparison. Differential gene enrichment analysis revealed that biological processes enriched in the Control group vs. HFD group and Control group vs. CTRP3 group were primarily related to mitochondrial organization, purine compound metabolism, purine nucleotide metabolism, purine ribonucleotide metabolism, nucleotide metabolism, ribonucleotide metabolism, ribose phosphate metabolism, and cellular respiration. Enrichment of KEGG signaling pathways mainly occurred in carbon metabolism, degradation of valine, leucine, and isoleucine, tricarboxylic acid (TCA) cycle, pyruvate metabolism, diabetic cardiomyopathy, ketone body metabolism, and glyoxylate and dicarboxylate metabolism.

In the context of myocardial lipotoxicity, lipid metabolism plays a crucial role. Lipid substances, such as triglycerides, cholesterol, and free fatty acids taken up by myocardial cells, are primarily utilized in mitochondria. The analysis results showed that differentially expressed genes in the Control group vs. HFD group and Control group vs. CTRP3 group were mainly concentrated in mitochondrial organization, ribonucleotide metabolism, energy production from organic compound oxidation, cellular respiration, metabolite and energy generation, potassium ion transport, and negative regulation of carboxylic acid biosynthesis. These processes are closely related to myocardial mitochondrial function, indicating that CTRP3 likely exerts its function in cardiac mitochondria, participating in the metabolic regulation of myocardial lipotoxicity.

Based on the above results, we propose that the shared interaction site of high-fat diet and CTRP3 might primarily be in the mitochondria. Myocardial mitochondria are the main sites for ATP production and energy exchange in the heart. Therefore, we further explored the potential relationship between myocardial mitochondria and the occurrence of myocardial lipotoxicity.

### 3.8 CTRP3 alleviates mitochondrial damage and autophagy in cardiomyocytes

Transmission electron microscopy revealed mitochondrial damage in cardiomyocytes following sustained high-fat diet stimulation, characterized by the disappearance of mitochondrial cristae and the formation of autophagosomes. Additionally, mitochondrial JC-1 staining and MitoTracker staining showed decreased mitochondrial membrane potential and reduced mitochondrial quantity in cardiomyocytes treated with palmitic acid (PA). However, overexpression of CTRP3 and exogenous supplementation with recombinant CTRP3 (rCTRP3) significantly improved the structure and function of cardiomyocyte mitochondria, reduced autophagosome formation, and increased ATP production. These results suggest that CTRP3 may participate in regulating mitochondrial autophagy to improve myocardial function.

In conjunction with the analysis of CTRP3's role in alleviating myocardial lipotoxicity and the RNA sequencing results, this study focused on the structure and function of mitochondria in cardiomyocytes, which are the primary sites for energy metabolism and ATP production in the heart. The study further investigated the effects of high-fat stimulation and CTRP3 intervention on cardiomyocyte mitochondria.

**Figure 8:**
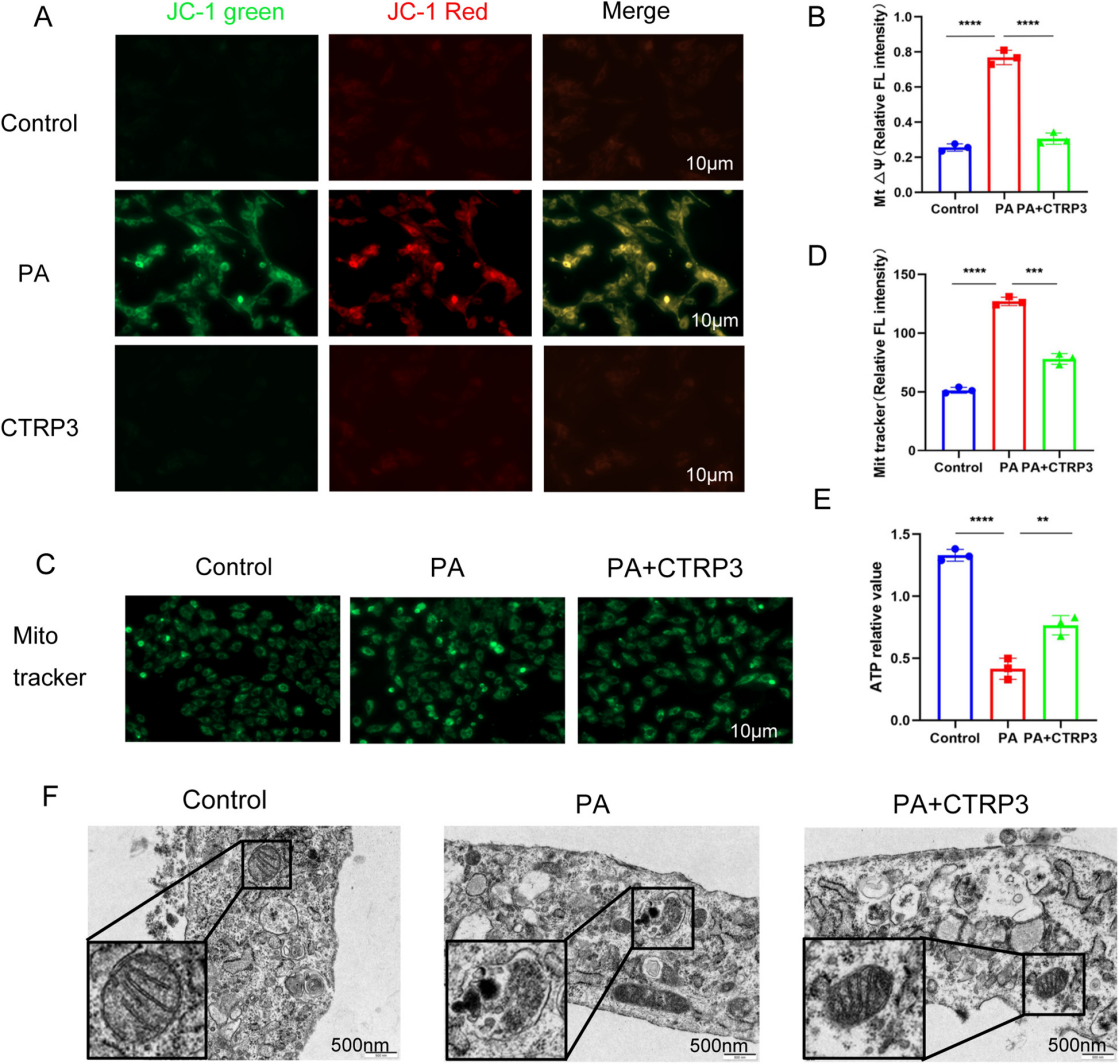
**(A,B)** JC-1 staining results demonstrate a decrease in mitochondrial membrane potential in myocardial cells of the PA group. **(C,D)** Mito-Tracker staining results show a reduction in mitochondrial number in the PA group. **(E)** PA intervention in myocardial cells leads to decreased ATP production, while CTRP3 intervention improves ATP generation. **(F)** Transmission electron microscopy reveals increased mitochondrial damage and autophagy in myocardial cells following PA intervention. CTRP3 can mitigate mitochondrial damage and autophagy in myocardial cells.

In this section, experiments were conducted using mitochondrial membrane potential detection (JC-1) and mitochondrial green fluorescence probe (MitoTracker Green) imaging to assess mitochondrial membrane potential and activity in H9c2 cardiomyocytes (Figure 8A–D). The results indicated that high-fat stimulation led to a decrease in mitochondrial membrane potential and enhanced mitochondrial activity in H9c2 cardiomyocytes. ATP detection revealed reduced ATP production in cardiomyocytes after high-fat stimulation, and transmission electron microscopy images showed ruptured mitochondrial double membranes and incomplete mitochondrial morphology. However, intervention with recombinant CTRP3 protein improved or alleviated mitochondrial double membrane structure, morphology, membrane potential damage, mitochondrial activity, and ATP production in cardiomyocytes (Figure 8E,F).

## 4 Discussion

Myocardial lipotoxicity is a pathological process characterized by myocardial cell damage resulting from dysregulated fatty acid metabolism, leading to lipid accumulation, inflammation, and oxidative stress (8, 24). During this process, abnormal fatty acid uptake and oxidation lead to excessive lipid accumulation within cells (24). This metabolic imbalance and mitochondrial dysfunction result in lipid accumulation, activation of inflammatory responses (25), and the initiation of cell death pathways, ultimately leading to myocardial injury (26, 27).

The various types of myocardial damage caused by lipotoxicity include myocardial cell death, abnormal calcium regulation within myocardial cells, mitochondrial damage (28), disruption of intercellular connections, and impaired myocardial contractile function (29). These injuries affect myocardial contractility and relaxation, ultimately leading to a decline in cardiac function (29–31). Additionally, myocardial lipotoxicity can induce changes in the microstructure of the myocardium. In the early stages of myocardial lipotoxicity, lipid accumulation within myocardial cells leads to increased cell volume (32, 33). As lipid accumulation progresses, small lipid droplets within the cells fuse into larger lipid bodies, damaging the structural integrity of myocardial cells. Concurrently, mitochondrial changes occur, such as a reduction in mitochondrial number and disruption of mitochondrial internal structure (34). These changes impair energy metabolism within myocardial cells, contributing to the decline in myocardial function.

In this study, we referred to some literature on the optimal dose of PA-induced lipotoxicity in cardiomyocytes, which suggested that 300–400 μM PA would induce lipotoxicity (35–37). However, during the cell experiment, we found that 300–400 μM PA caused severe acute damage to cardiomyocytes, with a large number of cardiomyocytes undergoing irreversible death. This damage does not conform to the chronic, long-term, and gradual accumulation process of lipotoxicity. In addition, we found that 50 μM PA would induce damage to cardiomyocytes, and the degree of damage increased with time, which is consistent with the chronic damage process of lipotoxicity. Therefore, in this study, we chose the condition of 50 μM PA stimulation of cardiomyocytes for 72 h to induce a lipotoxicity cell model.

In the study, the results show the comparison of echocardiographic results between ob/ob mice fed a high-fat diet (HFD) and those fed a standard diet (Figure 1B,C). At each identical time point, there were no significant differences in EF (ejection fraction) and FS (fractional shortening) values between the control group and the HFD group. However, when examining different time points, our study found that cardiac function in both the HFD and control groups significantly declined over time. Additionally, Figure 1D–F demonstrates that cardiomyocytes in the HFD group accumulated a large number of lipid droplets and exhibited disordered myocardial tissue structure. This indicates that although long-term HFD and standard diet seemingly do not cause significant differences or cardiac dysfunction in ob/ob mice at the macroscopic level, severe lipid droplet accumulation and subsequent myocardial tissue disarray and damage have already occurred at the microscopic level within cardiomyocytes. The results in Supplementary Figure S1A–C further confirm this observation.

Thus, macroscopic and microscopic changes in cardiac lipotoxicity do not occur synchronously. This phenomenon provides a novel perspective for further investigating the occurrence and underlying mechanisms of cardiac lipotoxicity. In this model, whether myocardial lipid accumulation directly affects cardiac function is gradually explored. We must consider the following question: Is cardiac macroscopic dysfunction always synchronous with microscopic cardiomyocyte damage? Must we only pay attention to changes at the cardiomyocyte level when cardiac EF values decline and severe heart failure occurs? Focusing on changes in lipid metabolism at the cardiomyocyte level in advance may offer a new approach for studying the chronic, long-term, and gradual accumulation process of cardiac lipotoxicity.

Therefore, the series of damages caused by myocardial lipotoxicity, along with the microstructural changes in the myocardium, collectively contribute to the decline in cardiac function. Therefore, controlling imbalances in fatty acid metabolism and preventing myocardial lipotoxicity are crucial for preventing cardiovascular diseases and reducing the risk of heart attacks and fatalities.

Myocardial lipotoxicity is a key factor in the development of cardiovascular diseases and has garnered widespread attention. Some studies focus on inhibiting lipid accumulation and regulating lipid metabolism to mitigate myocardial cell damage (27). Other research is centered on exploring potential drugs or molecular targets to improve myocardial function and prevent the progression of cardiovascular diseases (38). Further studies will help elucidate the pathogenesis of myocardial lipotoxicity, develop new therapeutic strategies, and provide more options for the prevention and treatment of cardiovascular diseases.

Moreover, key molecules currently involved in myocardial lipotoxicity research include fatty acid transport proteins (39), fatty acid oxidation enzymes (40), PPAR (41), AMPK (42, 43), JNK (44), and mTOR (45), among others. These molecules participate in lipid metabolism and regulation mechanisms and are associated with the occurrence and progression of myocardial lipotoxicity. The JNK (c-Jun N-terminal kinase) and MAPK (mitogen-activated protein kinase) pathways (46–48) also play crucial roles in lipid-induced myocardial inflammation. These pathways can respond to various stimuli, including oxidative stress and inflammatory factors, and subsequently activate multiple inflammation-related genes. Although the JNK and MAPK pathways are important in inflammation, this study prioritized the downstream inflammatory cytokines IL-6, IL-1β, and TNF-α (The improvement of IL1βR1 and IL1α levels under high-fat conditions by CTRP3 treatment was also verified in Supplementary Figure S6). In future research, we plan to evaluate the JNK and MAPK pathways to gain a more comprehensive understanding of CTRP3's anti-inflammatory mechanisms. This will help to elucidate whether CTRP3 mitigates inflammation through the synergistic action of multiple pathways.

CTRP3 has been demonstrated to play a significant role in various metabolic processes (19, 22, 49), including the regulation of lipid metabolism and mitochondrial function. By enhancing mitochondrial function, CTRP3 may promote fatty acid oxidation, thereby reducing myocardial lipid accumulation. Mitochondria are central to cellular energy metabolism, and their dysfunction is associated with a variety of cardiovascular diseases. Restoring mitochondrial function and ATP production is crucial for maintaining normal cardiac function.

CTRP3 has protective and valuable effects in improving PA-induced lipotoxicity in cardiomyocytes. There are also some literatures (49–51) that can support my research results. Moreover, it is currently unclear whether CTRP3 doses reflect the physiological or supraphysiological levels observed under metabolic disease conditions. This is a direction worthy of in-depth exploration and research. The goal of our research is also to find the critical value of PA between physiological and supraphysiological levels in cardiomyocytes. In the chronic metabolic lipotoxicity process of cardiomyocytes, lipids accumulate gradually and cause damage. Macroscopic changes, such as cardiac LVEF values, may not be observed at the beginning. However, microscopic changes, such as mitochondrial damage in cardiomyocytes, may have already occurred.

In the study, we also find that CPT1A (52, 53) is a key enzyme in fatty acid oxidation, responsible for transporting long-chain fatty acids into the mitochondrial matrix for oxidation. Increased activity of CPT1A is typically associated with enhanced fatty acid oxidation. Similarly, HADH, a key enzyme in the β-oxidation of fatty acids (54), also indicates enhanced fatty acid oxidation when its activity is increased. By examining the expression levels of CPT1A and HADH, we can assess whether CTRP3 reduces myocardial lipid accumulation by enhancing mitochondrial function and fatty acid oxidation. Further investigation into the mechanisms by which CTRP3 affects mitochondrial function and fatty acid oxidation, including its regulation of CPT1A and HADH expression, is warranted. In future, we can measure the protein and mRNA levels of CPT1A and HADH, as well as other markers related to mitochondrial function (such as mitochondrial membrane potential and ATP production), to comprehensively evaluate the mechanisms of action of CTRP3. Additionally, combining lipidomics and metabolomics analyses will provide a deeper understanding of the effects of CTRP3 on myocardial lipid metabolism and mitochondrial function.

In summary, in-depth research into the mechanisms of action of these molecules may help us better understand the pathogenesis of myocardial lipotoxicity and develop more effective treatment strategies (3, 8). In terms of preventing and treating myocardial lipotoxicity, diet and exercise are crucial. Proper diet and exercise can help control fat intake and metabolism (26), thereby reducing the metabolic burden on the body and improving cardiovascular health.

## Data Availability

The datasets presented in this study can be found in online repositories. The names of the repository/repositories and accession number(s) can be found in the article/Supplementary Material.

## References

[1] Jahangir E De Schutter A Lavie CJ. The relationship between obesity and coronary artery disease. Transl Res. (2014) 164:336–44. 10.1016/j.trsl.2014.03.010 24726461

[2] Iacobellis G. Epicardial fat links obesity to cardiovascular diseases. Prog Cardiovasc Dis. (2023) 78:27–33. 10.1016/j.pcad.2023.04.006 37105279

[3] Ren J Wu NN Wang S Sowers JR Zhang Y. Obesity cardiomyopathy: evidence, mechanisms, and therapeutic implications. Physiol Rev. (2021) 101:1745–807. 10.1152/physrev.00030.2020 33949876 PMC8422427

[4] Goldsborough E Osuji N Blaha MJ. Assessment of cardiovascular disease risk. Endocrinol Metab Clin North Am. (2022) 51:483–509. 10.1016/j.ecl.2022.02.005 35963625

[5] Szuszkiewicz-Garcia MM Davidson JA. Cardiovascular disease in diabetes mellitus. Endocrinol Metab Clin North Am. (2014) 43:25–40. 10.1016/j.ecl.2013.09.001 24582090

[6] Soppert J Lehrke M Marx N Jankowski J Noels H. Lipoproteins and lipids in cardiovascular disease: from mechanistic insights to therapeutic targeting. Adv Drug Deliv Rev. (2020) 159:4–33. 10.1016/j.addr.2020.07.019 32730849

[7] Bertero E Maack C. Metabolic remodelling in heart failure. Nat Rev Cardiol. (2018) 15:457–70. 10.1038/s41569-018-0044-6 29915254

[8] Sletten AC Peterson LR Schaffer JE. Manifestations and mechanisms of myocardial lipotoxicity in obesity. J Intern Med. (2018) 284:478–91. 10.1111/joim.12728 29331057 PMC6045461

[9] Kok BPC Brindley DN. Myocardial fatty acid metabolism and lipotoxicity in the setting of insulin resistance. Heart Fail Clin. (2012) 8:643–61. 10.1016/j.hfc.2012.06.008 22999246

[10] Tong M Saito T Zhai P Oka S-I Mizushima W Nakamura M Mitophagy is essential for maintaining cardiac function during high fat diet-induced diabetic cardiomyopathy. Circ Res. (2019) 124:1360–71. 10.1161/CIRCRESAHA.118.314607 30786833 PMC6483841

[11] Levin MC Andersson L Borén J. Cardiomyocytes, sphingolipids and cardio myotoxicity. Curr Opin Lipidol. (2023) 34:180–8. 10.1097/MOL.0000000000000829 37431304

[12] Schulze PC. Myocardial lipid accumulation and lipotoxicity in heart failure. J Lipid Res. (2009) 50:2137–8. 10.1194/jlr.R001115 19687505 PMC2759818

[13] Leggat J Bidault G Vidal-Puig A. Lipotoxicity: a driver of heart failure with preserved ejection fraction? Clin Sci. (2021) 135:2265–83. 10.1042/CS20210127 PMC8543140 34643676

[14] Mukherjee AG Renu K Gopalakrishnan AV Jayaraj R Dey A Vellingiri B RETRACTED: epicardial adipose tissue and cardiac lipotoxicity: a review. Life Sci. (2023) 328:121913. 10.1016/j.lfs.2023.121913 37414140

[15] Li Y Wright GL Peterson JM. C1q/TNF-related protein 3 (CTRP3) function and regulation. Compr Physiol. (2017) 7:863–78. 10.1002/j.2040-4603.2017.tb00770.x 28640446 PMC5756469

[16] Yang Y Li Y Ma Z Jiang S Fan C Hu W A brief glimpse at CTRP3 and CTRP9 in lipid metabolism and cardiovascular protection. Prog Lipid Res. (2016) 64:170–7. 10.1016/j.plipres.2016.10.001 27743997

[17] Guo B Zhuang T Xu F Lin X Li F Shan S-K New insights into implications of CTRP3 in obesity, metabolic dysfunction, and cardiovascular diseases: potential of therapeutic interventions. Front Physiol. (2020) 11:570270. 10.3389/fphys.2020.570270 33343381 PMC7744821

[18] Peterson JM Wei Z Wong GW. C1q/TNF-related protein-3 (CTRP3), a novel adipokine that regulates hepatic glucose output. J Biol Chem. (2010) 285:39691–701. 10.1074/jbc.M110.180695 20952387 PMC3000950

[19] Yi W Sun Y Yuan Y Lau WB Zheng Q Wang X C1q/tumor necrosis factor-related protein-3, a newly identified adipokine, is a novel antiapoptotic, proangiogenic, and cardioprotective molecule in the ischemic mouse heart. Circulation. (2012) 125:3159–69. 10.1161/CIRCULATIONAHA.112.099937 22653084 PMC3391311

[20] Si Y Fan W Sun L. A review of the relationship between CTRP family and coronary artery disease. Curr Atheroscler Rep. (2020) 22:22. 10.1007/s11883-020-00840-0 32468164 PMC7256102

[21] Wei W-Y Ma Z-G Zhang N Xu S-C Yuan Y-P Zeng X-F Overexpression of CTRP3 protects against sepsis-induced myocardial dysfunction in mice. Mol Cell Endocrinol. (2018) 476:27–36. 10.1016/j.mce.2018.04.006 29655602

[22] Ma Z-G Yuan Y-P Xu S-C Wei W-Y Xu C-R Zhang X CTRP3 attenuates cardiac dysfunction, inflammation, oxidative stress and cell death in diabetic cardiomyopathy in rats. Diabetologia. (2017) 60:1126–37. 10.1007/s00125-017-4232-4 28258411

[23] Lin J Liu Q Zhang H Huang X Zhang R Chen S C1q/Tumor necrosis factor-related protein-3 protects macrophages against LPS-induced lipid accumulation, inflammation and phenotype transition via PPAR*γ* and TLR4-mediated pathways. Oncotarget. (2017) 8:82541–57. 10.18632/oncotarget.19657 29137283 PMC5669909

[24] Borradaile NM Schaffer JE. Lipotoxicity in the heart. Curr Hypertens Rep. (2005) 7:412–7. 10.1007/s11906-005-0035-y 16386196

[25] Wenzl FA Ambrosini S Mohammed SA Kraler S Lüscher TF Costantino S Inflammation in metabolic cardiomyopathy. Front Cardiovasc Med. (2021) 8:742178. 10.3389/fcvm.2021.742178 34671656 PMC8520939

[26] Schulze PC Drosatos K Goldberg IJ. Lipid use and misuse by the heart. Circ Res. (2016) 118:1736–51. 10.1161/CIRCRESAHA.116.306842 27230639 PMC5340419

[27] Wende AR Symons JD Abel ED. Mechanisms of lipotoxicity in the cardiovascular system. Curr Hypertens Rep. (2012) 14:517–31. 10.1007/s11906-012-0307-2 23054891 PMC3491122

[28] Yan M Li Y Luo Q Zeng W Shao X Li L Mitochondrial damage and activation of the cytosolic DNA sensor cGAS-STING pathway lead to cardiac pyroptosis and hypertrophy in diabetic cardiomyopathy mice. Cell Death Discov. (2022) 8:258. 10.1038/s41420-022-01046-w 35538059 PMC9091247

[29] Chumakova G Gritsenko O Gruzdeva O Dyleva Y. Analysis of probable lipotoxic damage and myocardial fibrosis in epicardial obesity. Aging. (2021) 13:14806–15. 10.18632/aging.203148 34088886 PMC8221350

[30] Costantino S Akhmedov A Melina G Mohammed SA Othman A Ambrosini S Obesity-induced activation of JunD promotes myocardial lipid accumulation and metabolic cardiomyopathy. Eur Heart J. (2019) 40(12):997–1008. 10.1093/eurheartj/ehy903 30629164

[31] Jiménez-González S Marín-Royo G Jurado-López R Bartolomé MV Romero-Miranda A Luaces M The crosstalk between cardiac lipotoxicity and mitochondrial oxidative stress in the cardiac alterations in diet-induced obesity in rats. Cells. (2020) 9:451. 10.3390/cells9020451 32079154 PMC7072852

[32] Gao H Feng X-J Li Z-M Li M Gao S He Y-H Downregulation of adipose triglyceride lipase promotes cardiomyocyte hypertrophy by triggering the accumulation of ceramides. Arch Biochem Biophys. (2015) 565:76–88. 10.1016/j.abb.2014.11.009 25436917

[33] Karpanen T Bry M Ollila HM Seppänen-Laakso T Liimatta E Leskinen H Overexpression of vascular endothelial growth factor-B in mouse heart alters cardiac lipid metabolism and induces myocardial hypertrophy. Circ Res. (2008) 103:1018–26. 10.1161/CIRCRESAHA.108.178459 18757827 PMC2762522

[34] Law BA Liao X Moore KS Southard A Roddy P Ji R Lipotoxic very-long-chain ceramides cause mitochondrial dysfunction, oxidative stress, and cell death in cardiomyocytes. FASEB J. (2018) 32:1403–16. 10.1096/fj.201700300R 29127192 PMC5892719

[35] Fonseka O Raja R Ross C Gare SR Zhang J Hille SS XBP1s-EDEM2 prevents the onset and development of HFpEF by ameliorating cardiac lipotoxicity. Circulation. (2025). 10.1161/CIRCULATIONAHA.124.072194 40130322 PMC12124211

[36] Zhang H Xu T Mei X Zhao Q Yang Q Zeng X PINK1 modulates Prdx2 to reduce lipotoxicity-induced apoptosis and attenuate cardiac dysfunction in heart failure mice with a preserved ejection fraction. Clin Transl Med. (2025) 15:e70166. 10.1002/ctm2.70166 39763059 PMC11705485

[37] He Y Li S Jiang L Wu K Chen S Su L Palmitic acid accelerates endothelial cell injury and cardiovascular dysfunction via palmitoylation of PKM2. Adv Sci. (2025) 12:e2412895. 10.1002/advs.202412895 PMC11791964 39665133

[38] Pulinilkunnil T Kienesberger PC Nagendran J Waller TJ Young ME Kershaw EE Myocardial adipose triglyceride lipase overexpression protects diabetic mice from the development of lipotoxic cardiomyopathy. Diabetes. (2013) 62:1464–77. 10.2337/db12-0927 23349479 PMC3636613

[39] Chiu H-C Kovacs A Blanton RM Han X Courtois M Weinheimer CJ Transgenic expression of fatty acid transport protein 1 in the heart causes lipotoxic cardiomyopathy. Circ Res. (2005) 96:225–33. 10.1161/01.RES.0000154079.20681.B9 15618539

[40] Haffar T Bérubé-Simard F Bousette N. Impaired fatty acid oxidation as a cause for lipotoxicity in cardiomyocytes. Biochem Biophys Res Commun. (2015) 468:73–8. 10.1016/j.bbrc.2015.10.162 26546819

[41] Duerr GD Heinemann JC Arnoldi V Feisst A Kley J Ghanem A Cardiomyocyte specific peroxisome proliferator-activated receptor-α overexpression leads to irreversible damage in ischemic murine heart. Life Sci. (2014) 102:88–97. 10.1016/j.lfs.2014.03.019 24657893

[42] Yang Z Chen Y Yan Z Xu TT Wu X Pi A Inhibition of TLR4/MAPKs pathway contributes to the protection of salvianolic acid a against lipotoxicity-induced myocardial damage in cardiomyocytes and obese mice. Front Pharmacol. (2021) 12:627123. 10.3389/fphar.2021.627123 33762947 PMC7982403

[43] Zhang W Lu J Wang Y Sun P Gao T Xu N Canagliflozin attenuates lipotoxicity in cardiomyocytes by inhibiting inflammation and ferroptosis through activating AMPK pathway. Int J Mol Sci. (2023) 24(1):858. 10.3390/ijms24010858 36614295 PMC9821072

[44] Mangali S Bhat A Udumula MP Dhar I Sriram D Dhar A. Inhibition of protein kinase R protects against palmitic acid-induced inflammation, oxidative stress, and apoptosis through the JNK/NF-kB/NLRP3 pathway in cultured H9C2 cardiomyocytes. J Cell Biochem. (2019) 120:3651–63. 10.1002/jcb.27643 30259999

[45] Wang L Zhao D Tang L Li H Liu Z Gao J Soluble epoxide hydrolase deficiency attenuates lipotoxic cardiomyopathy via upregulation of AMPK-mTORC mediated autophagy. J Mol Cell Cardiol. (2021) 154:80–91. 10.1016/j.yjmcc.2020.12.013 33378686 PMC8068623

[46] Wang X Liu R Liu D. The role of the MAPK signaling pathway in cardiovascular disease: pathophysiological mechanisms and clinical therapy. Int J Mol Sci. (2025) 26(6):2667. 10.3390/ijms26062667 40141309 PMC11942496

[47] Bai B Ji Z Wang F Qin C Zhou H Li D CTRP12 ameliorates post-myocardial infarction heart failure through down-regulation of cardiac apoptosis, oxidative stress and inflammation by influencing the TAK1-p38 MAPK/JNK pathway. Inflamm Res. (2023) 72:1375–90. 10.1007/s00011-023-01758-4 37382682

[48] Zhang Z Yang Z Wang S Wang X Mao J. Targeting MAPK-ERK/JNK pathway: a potential intervention mechanism of myocardial fibrosis in heart failure. Biomed Pharmacother. (2024) 173:116413. 10.1016/j.biopha.2024.116413 38461687

[49] Song Y Zhang Y Wan Z Pan J Gao F Li F CTRP3 alleviates myocardial ischemia/reperfusion injury in mice through activating LAMP1/JIP2/JNK pathway. Int Immunopharmacol. (2022) 107:108681. 10.1016/j.intimp.2022.108681 35278832

[50] Mu Y Yin T-L Yin L Hu X Yang J. CTRP3 attenuates high-fat diet-induced male reproductive dysfunction in mice. Clin Sci. (2018) 132:883–99. 10.1042/CS20180179 29572383

[51] Liu Y Wu P Xu X Shen T Wang X Liu Y C1q/TNF-related protein 3 alleviates heart failure via attenuation of oxidative stress in myocardial infarction rats. Peptides. (2023) 163:170980. 10.1016/j.peptides.2023.170980 36842629

[52] Actis Dato V Lange S Cho Y. Metabolic flexibility of the heart: the role of fatty acid metabolism in health, heart failure, and cardiometabolic diseases. Int J Mol Sci. (2024) 25:1211. 10.3390/ijms25021211 38279217 PMC10816475

[53] Tian X Chen X Jiang Q Sun Q Liu T Hong Y Notoginsenoside R1 ameliorates cardiac lipotoxicity through AMPK signaling pathway. Front Pharmacol. (2022) 13:864326. 10.3389/fphar.2022.864326 35370720 PMC8968201

[54] Foomani FH Jarzembowski JA Mostaghimi S Mehrvar S Kumar SN Ranji M. Optical metabolic imaging of mitochondrial dysfunction on HADH mutant newborn rat hearts. IEEE J Transl Eng Health Med. (2021) 9:1. 10.1109/JTEHM.2021.3104966 34462673 PMC8396955

