# Supplementary material for: CTRP3 attenuates myocardial lipotoxicity via suppression of lipid accumulation, inflammation, apoptosis, and mitochondrial oxidative stress
Source: Front Cardiovasc Med. 2025 May 13;12:1575929. doi: 10.3389/fcvm.2025.1575929 (PMC12106403; doi:10.3389/fcvm.2025.1575929)

Real-time PCR analysis was performed using the following primers:

Supplementary Table.1

Gene	Primer sequence
Rat TNF-α	F：ATGGGCTCCCTCTCATCAGT<br>R：TCCAGTGAGTTCCGAAAGCC
Rat IL-1β	F：AGGCTGACAGACCCCAAAAG<br>R：CAGGGAGGGAAACACACGTT
Rat IL-6	F：CACTTCACAAGTCGGAGGCT<br>R：AGCACACTAGGTTTGCCGAG
Rat GAPDH	F: GAGACAGCCGCATCTTCTTG<br>R: GAGACAGCCGCATCTTCTTG
Rat Bax	F: CACGTCTGCGGGGAGTCA<br>R: TAGGAAAGGAGGCCATCCCA
Rat Bcl_2	F: GAGGGGCTACGAGTGGGATA<br>R: GCATGCTGGGGCCATATAGT
Rat SOD1	F: TAACTGAAGGCGAGCATGGG<br>R: TCCCAATCACACCACAAGCC
Rat SOD2	F: ACCGAGGAGAAGTACCACGA<br>R: CCTGAACCTTGGACTCCCAC
Rat Nppa	F: CCTGGACTGGGGAAGTCAAC<br>R: GCAGCTCCAGGAGGGTATTC
Rat Nppb	F: TCCTTAATCTGTCGCCGCTG<br>R: GGCGCTGTCTTGAGACCTAA
Rat FATP4	F：CGCTGGAAAGGGGAGAATGT<br>R：CACTTTGGCAGCTTTGGACC
Rat FABP4	F：GCGTAGAAGGGGACTTGGTC<br>R：CGAATTCCACGCCCAGTTTG
Rat PPARα	F：GTCCTCTGGTTGTCCCCTTG<br>R：TGGGGAGAGAGGACAGATGG

Rat PPAR delta	F：GATGAGGACAAACCCACGGT<br>R：CATGCACGCTGATCTCGTTG
Rat VLDLR	F：GTGCAGCTGGGTTTGAACTG<br>R：GCTGCAATGTCAGCATCGAG
Rat ACSL1	F：GGCAGTTCGTGGATTGCAG<br>R：CATTGCTCCTTTGGGGTTGC
Rat LPL	F：TCCCAATGGAGGCACTTTCC<br>R：TCTGACCAGCGGAAGTAGGA
Rat FoxO1	F：CTCAGGTGGTGGAGACCGA<br>R：GAGCTGGTTCGAGGACGAAA
Rat ABCA1	F：GCGACCATGAAAGTGACACG<br>R：AAACCCTTTCTACTCCGCCG
Rat ABCG1	F：CTTACTCCGTACCCGAGGGA<br>R：TCCTTGACCATCTCCCGTCT

Supplementary Figure.1 A Myocardial Oil Red O Staining; B Myocardial Hematoxylin and Eosin Staining; C Myocardial Masson's Trichrome Staining. The Oil Red O staining result suggests that high-fat diet leads to significant lipid droplet accumulation in myocardial tissue. And

the HE and Masson result indicates that a high-fat diet leads to structural disarray and cardiac fibrosis in myocardial tissue.

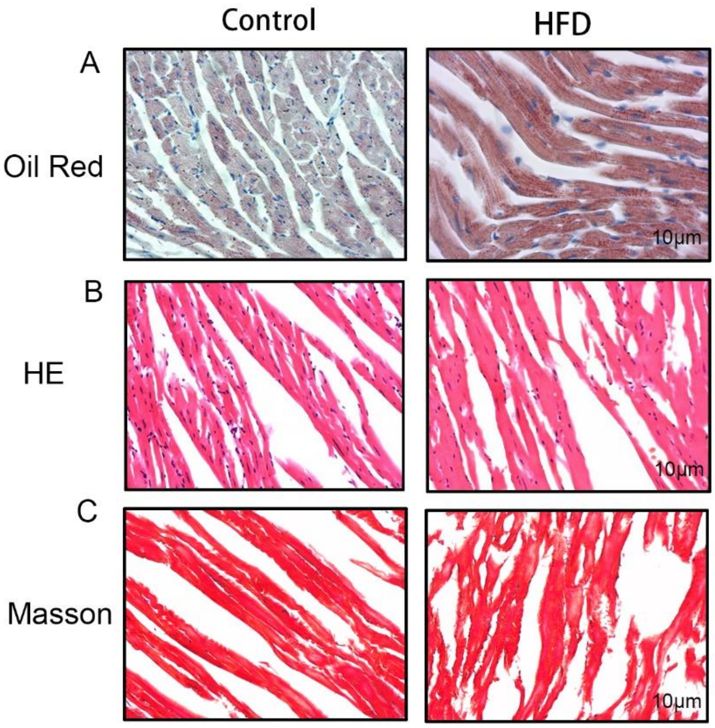

Supplementary Figure.2 A The damage of myocardial cells treated with different concentrations of PA. B The CCK8 results indicate that treating myocardial cells with PA for 24 hours leads to a gradual decrease in myocardial cell viability with increasing PA concentration. C CCK8 results suggest that treatment of myocardial cells with 50μm PA leads to a decrease in myocardial cell viability with increasing treatment time.

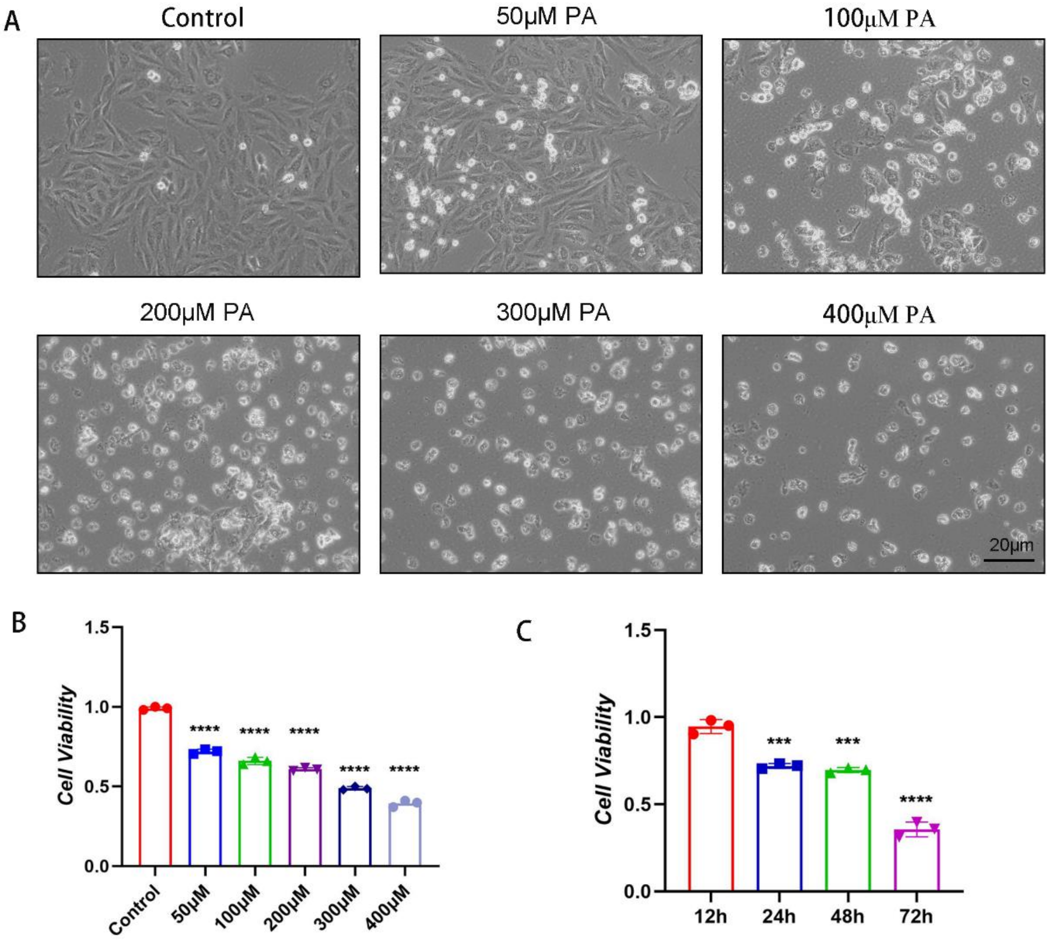

Supplementary Figure.3 A-B The flow cytometry results indicate that with the increase of PA concentration, the oxidative stress level in myocardial cells gradually rises.

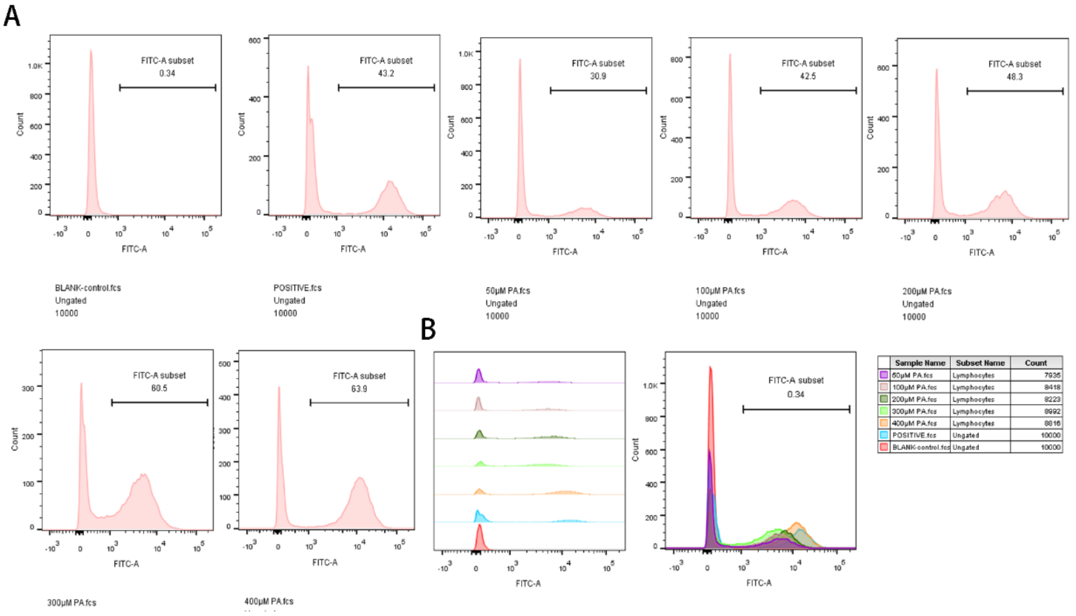

Supplementary Figure.4 A After adding CTRP3 in H9C2 or NRCM myocardial cells, the viability and condition of myocardial cells significantly improved compared to the PA group. B Treating myocardial cells with 50μm PA for 24 hours, adding CTRP3 at different concentrations make improvement in myocardial cell viability.

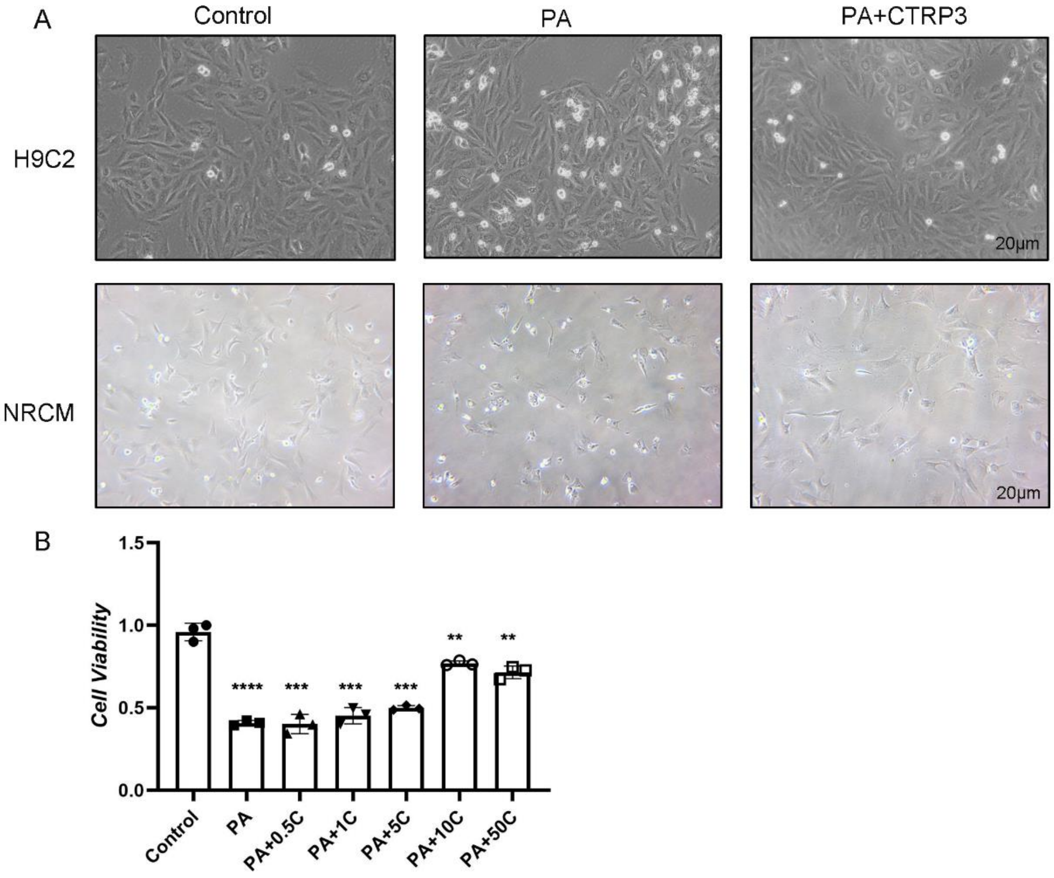

Supplementary Figure.5 A-B Treatment of myocardial cells with 50μm PA for 24 hours, followed by the addition of 10μg/ml CTRP3, resulted in reduced apoptosis damage in myocardial cells compared to the PA group. C-D Treating myocardial cells with 50μm PA for 24 hours, followed by the addition of 10μg/ml CTRP3, resulted in a decrease in oxidative stress levels in myocardial cells compared to the PA group.

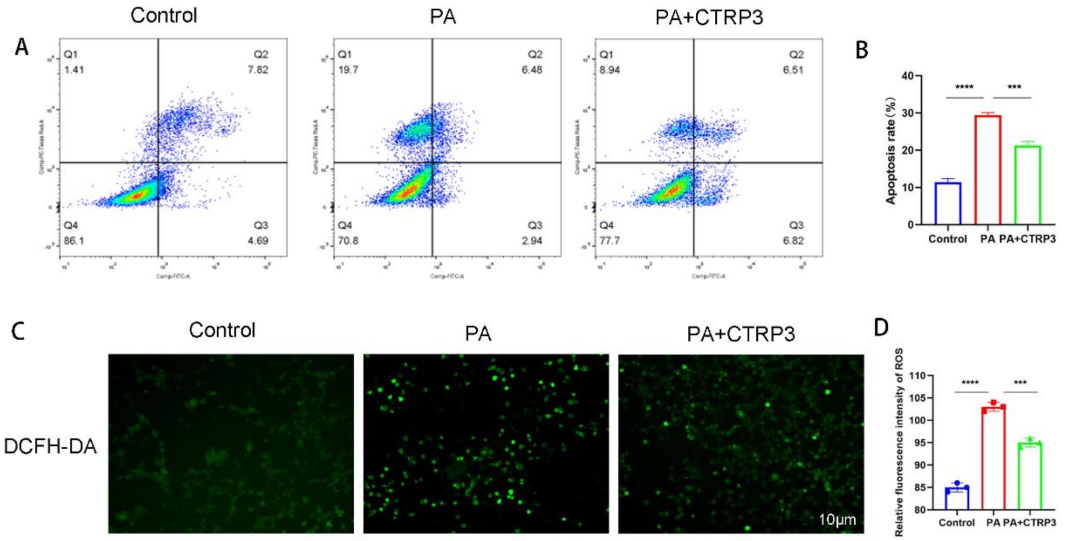

Supplementary Figure.6 A-B: PCR results indicate that CTRP3 can reduce the mRNA levels of the inflammatory factors IL1βR1 and IL1α in cardiomyocytes after PA treatment and in high-fat diet (HFD) conditions. C-D: ELISA results indicate that CTRP3 can reduce the levels of the inflammatory factors IL1βR1 and IL1α in the supernatant of cardiomyocytes after PA treatment and in serum after a high-fat diet.

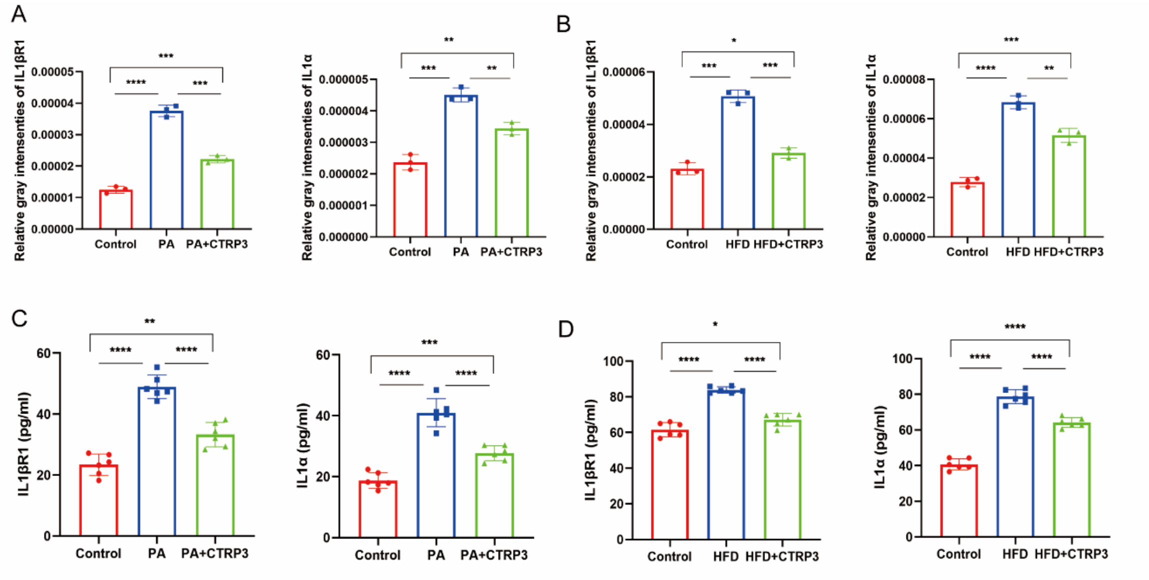

Supplement: Supplementary file 1 [file Datasheet1.pdf]
